# Identification of novel genes regulating the development of the palate

**DOI:** 10.1002/dvdy.70066

**Published:** 2025-08-02

**Authors:** Ashwin Bhaskar, Sophie Astrof

**Affiliations:** ^1^ School of Arts and Sciences Honors Program Rutgers University New Brunswick New Jersey USA; ^2^ Department of Cell Biology and Molecular Medicine Cardiovascular Research Institute, Rutgers Biomedical and Health Sciences Newark New Jersey USA

**Keywords:** cleft palate, eye, International Mouse Phenotyping Consortium, micro‐computed tomography, palate

## Abstract

**Background:**

The International Mouse Phenotyping Consortium (IMPC) has generated thousands of knockout mouse lines, many of which exhibit embryonic or perinatal lethality. Using micro‐computed tomography (micro‐CT), the IMPC has created and publicly released three‐dimensional image data sets of embryos from these lethal and subviable lines. In this study, we leveraged this data set to screen homozygous null mutants for anomalies in secondary palate development. We analyzed optical sections from 2987 embryos at embryonic days E15.5 and E18.5, representing 484 homozygous mutant lines.

**Results and Conclusions:**

Our analysis identified 44 novel genes implicated in palatogenesis. Gene set enrichment analysis highlighted biological processes and pathways relevant to palate development and uncovered 18 genes jointly regulating the development of the eye and the palate. These findings present a valuable resource for further research, offer novel insights into the molecular mechanisms underlying palatogenesis, and provide important context for understanding the etiology of rare human congenital disorders involving malformations of the palate and other organs.

## Key Findings

- Identified 44 novel genes regulating palatogenesis identified 18 genes jointly regulating the development of the eye and the palate.

## 1 INTRODUCTION

Cleft palate with or without a cleft lip (known as orofacial clefts) is the most prevalent craniofacial birth defect, affecting approximately 1 in 800 babies annually.^1^ There is a significant difference in the prevalence of orofacial clefting among different ethnic groups: the greatest prevalence was found among Native Americans at 3.6 per 1000 births, followed by Japanese and Chinese populations, with a prevalence of 2.1 and 1.7 per 1000 births, respectively, while the lowest prevalence is among African‐Americans, at approximately 0.3 per 1000 births.^2^ Children born with this disorder have difficulties with feeding and speech when untreated. In developed countries, orofacial clefts are surgically treated early in childhood to close the clefts.^2^ Despite the treatment, some affected individuals may still experience negative psychological, psychosocial, and financial hardships.^3^

Palate formation proceeds in multiple phases.^4^, ^5^ During the early stages of palate development, multi‐potent neural crest (NC)‐derived cells migrate to populate the first branchial arch, which subsequently gives rise to the maxillary and mandibular prominences. The secondary palate, the tissue that separates the nasal and oral cavities, forms when the paired outgrowths from the maxillary processes fuse at the midline and then with the primary palate, which is derived from the frontonasal prominence. In humans, palatal development takes place between Weeks 4 and 10 of gestation.^5^, ^6^ Due to the distinct developmental processes underlying the formation of the primary and secondary palate, several types of clefting are observed in patients and vertebrate model organisms: isolated cleft lip, isolated cleft palate, or clefting can be present both in the lip and palate.^6^, ^7^

Mice are an established model for studying palate formation and clefting due to similarities in palate development between mice and humans.^7^ In mice, the growth of paired palatal shelves from the maxillary processes can be observed at embryonic day (E) 11.5. These processes undergo a dramatic increase in size through the proliferation of NC‐derived mesenchyme and initially move downwards to be positioned on either side of the tongue by E13.5. The paired palatal shelves then elevate to become positioned horizontally above the tongue by E14.5. The fusion of palatal shelves at the midline begins soon afterward and results in the formation of a continuous shelf of the secondary palate. The fusion of the secondary palatal shelves is completed by E15.5, and the fusion of the primary and secondary palatal shelves is typically completed by E17.5.^8^

The causes of cleft lip and palate in the human population are not completely understood. Epidemiological studies, genetic analyses, and modeling of lip and palate clefting in mice indicated that both environmental factors and genetic mutations are at play.^4^, ^6^, ^7^ Among common mechanisms mediating palatal development in humans and mice are those regulated by Sonic Hedgehog (Shh) signaling.^9^, ^10^ Modeling studies in mice indicated that precise levels of Shh gene expression in the NC‐derived cells are essential for proper palate development, and both down‐regulation and up‐regulation of Shh in the NC‐derived cells cause clefting and other defects in facial development.^11^, ^12^ Additional players important for palate development include genes encoding secreted growth factors such as Bone Morphogenetic protein (BMPs), Wnts, and Fgfs, and transcriptional regulators such as the Pbx family of transcription factors and *Snail1*.^13^, ^14^, ^15^ However, much still needs to be learned. Therefore, it is critical to identify additional genes and their roles in palate development to broaden our understanding of mechanisms leading to the clefting of the lip and palate and to create more effective prognostic, diagnostic, and treatment options. To discover additional genes regulating the formation of the secondary palate, we examined images of mouse mutants generated and imaged by the International Mouse Phenotyping Consortium (IMPC) using micro‐computed tomography (micro‐CT) and looked for the presence of clefting in the secondary palate at E15.5 and E18.5 in coronal optical sections. Our analyses revealed novel genes regulating palatal development and provided new insights into the genetic etiology of rare human birth defects jointly affecting the development of the palate and other organs.

## 2 RESULTS AND DISCUSSION

**FIGURE 1:**
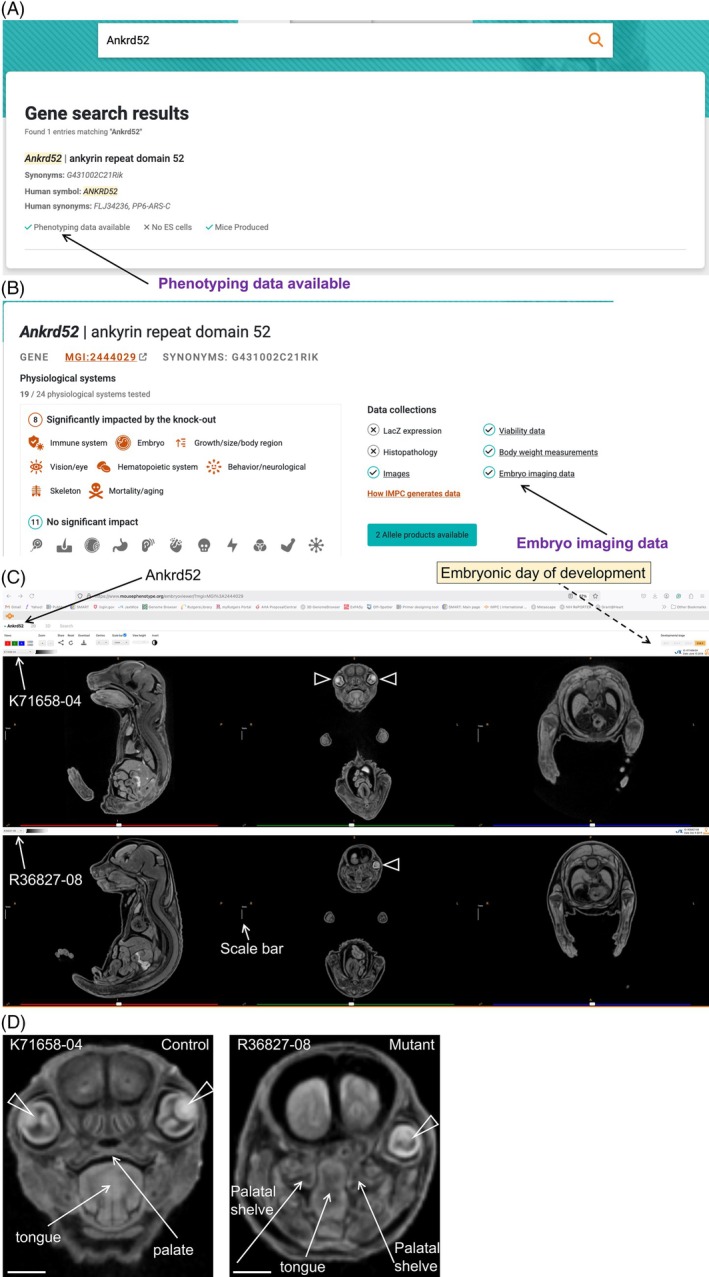
Accessing micro‐computed tomography (micro‐CT) image data on the International Mouse Phenotyping Consortium (IMPC) website. (A) To locate images of embryos, go to the IMPC website, https://www.mousephenotype.org/, and type the name of the gene of interest into the search box. In this example, the gene is Ankrd52. Follow the link to the phenotyping data (arrow). (B) To view micro‐CT images, follow the link to the embryo imaging data (arrow). (C) The page with images will contain a menu on the right to select the stage of embryonic development (dashed arrow). Images on the top are from control embryos. Images at the bottom are from mutants. To select the mutant of interest (e.g., E18.5 embryo # R36827‐08), select E18.5 developmental stage and then click on the dropdown arrow to the left of the mutant image (white arrows) and select the embryo of interest, in this case, # R36827‐08. Sagittal, coronal, and transverse optical sections can be examined by scrolling using the scroll bar below each image. The middle panel contains coronal optical sections; arrowheads point to the eyes. (D) Magnified coronal views of the control and Ankrd52 mutant heads are shown. Arrowheads point to the eyes. Note that this mutant has only one eye. Palatal shelves in the mutant have not lifted up, and the tongue protrudes between the shelves. Scale bars are 1 mm.

**FIGURE 2:**
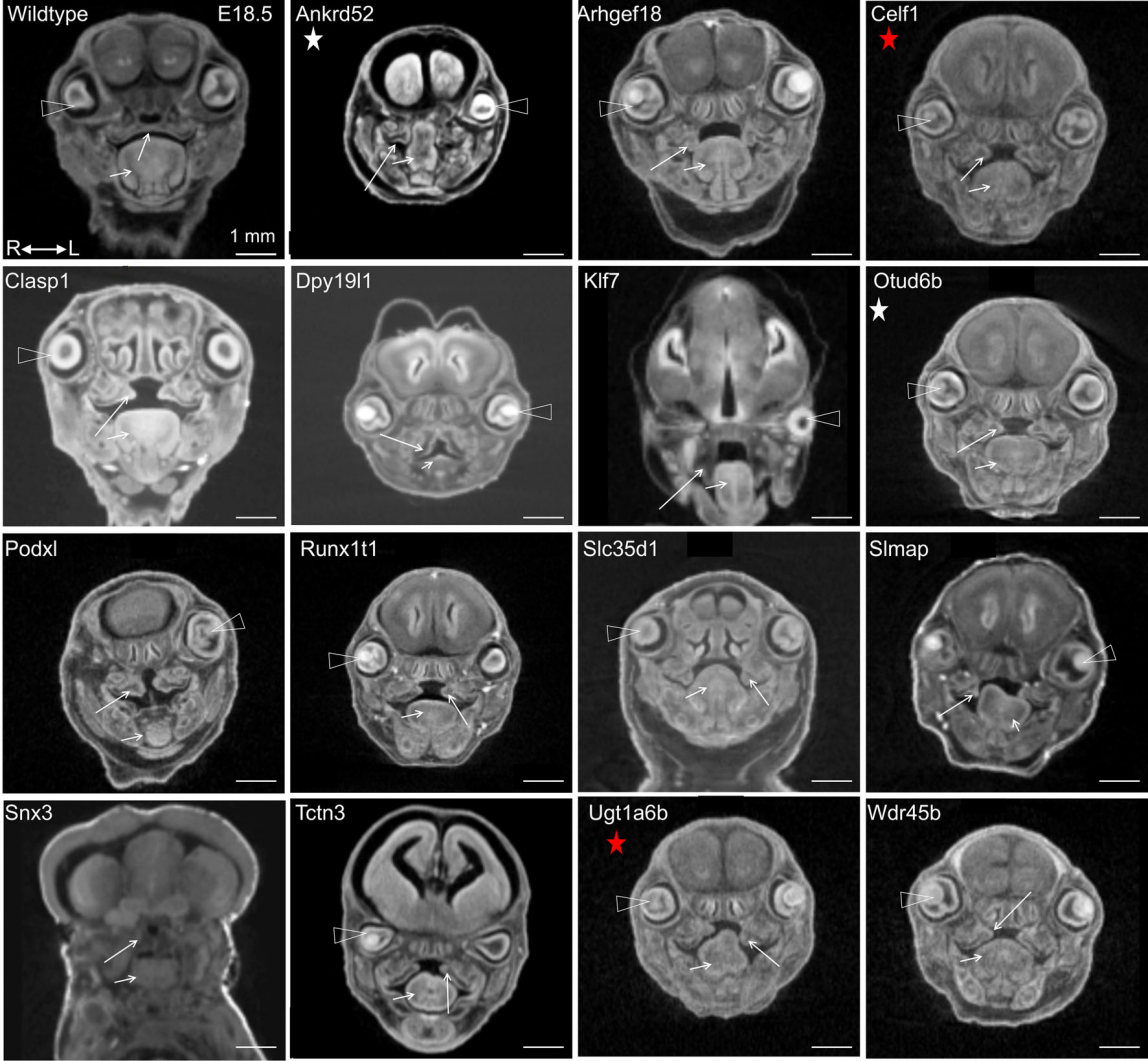
E18.5 mutants with cleft palate. Snapshots of coronal optical sections from micro‐computed tomography images at the eye level. Arrowheads mark eyes. Long arrows point at palatal shelves. Short arrows point at the tongue. Stars indicate mutant lines with palate and eye defects.Red stars mark mutant lines with eye defects shown in Figure 5. Table 2 contains additional information about the displayed embryos. All scale bars are 1 mm.

**FIGURE 3:**
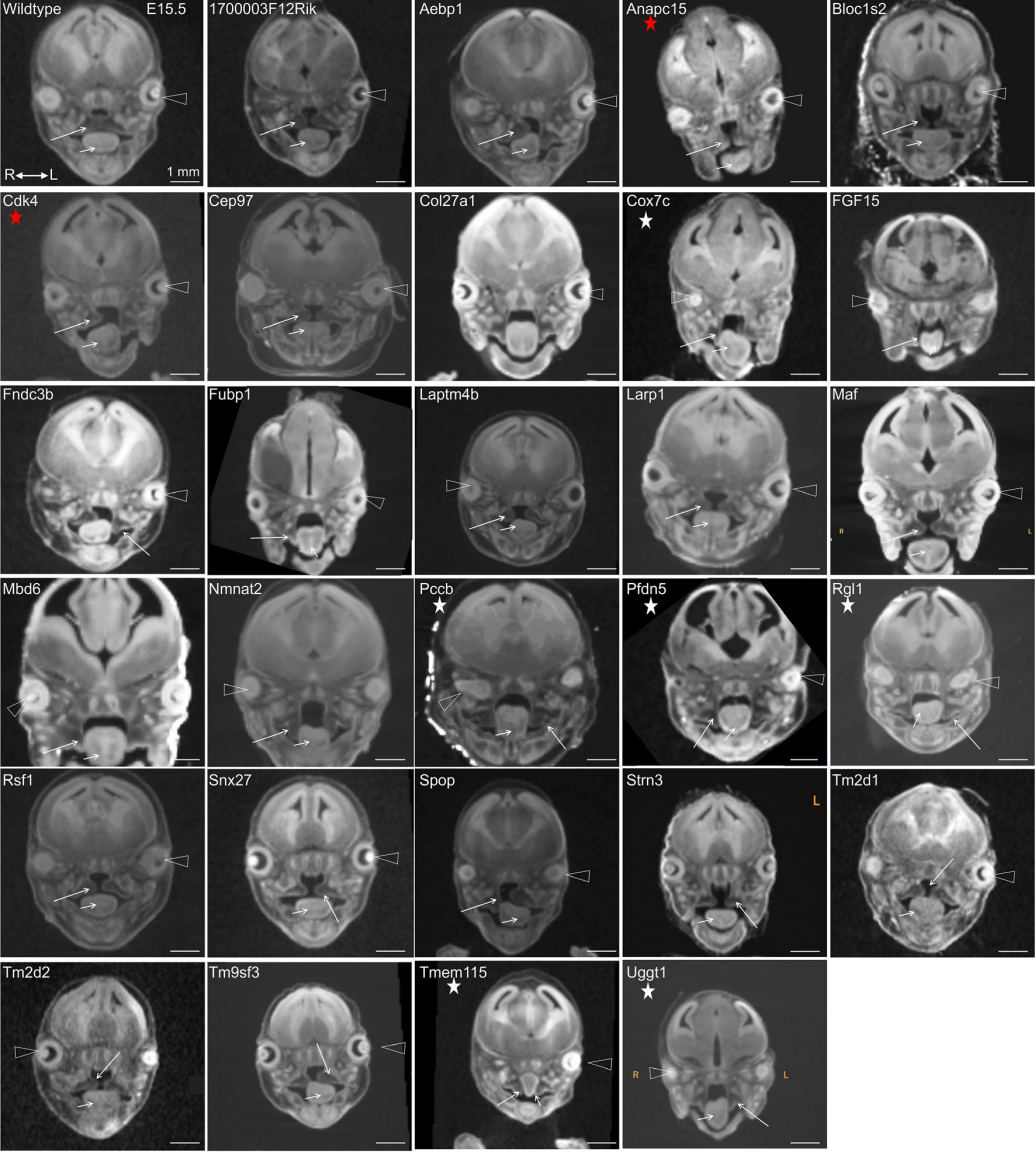
E15.5 mutants with cleft palate. Snapshots of coronal optical sections from micro‐computed tomography images were taken at eye level. Arrowheads mark eyes. Long arrows point at palatal shelves. Stars indicate mutants with palate and eye defects. Red stars mark mutant lines with eye defects shown in Figure 5. Short arrows point at the tongue. Table 3 contains additional information about the displayed embryos. All scale bars are 1 mm.

We examined the palatal morphology by analyzing coronal optical sections in 2987 embryos from 484 homozygous mutant lines generated and imaged by the IMPC (Figure 1; Table S1 reports cleft palate annotations of each examined embryo). Out of 484 homozygous mutant lines, 76 knockout lines contained embryos with cleft palates (Table 1, Figures 2 and 3). To determine which mutant lines represented novel cleft palate genes, we used gene ontology terms and the Mouse Genome Informatics (MGI) along with the cleft palate database (https://bioinfo.uth.edu/CleftGeneDB/) and manual searches. These analyses indicated that of 76 genes, homozygous‐null mutations in 44 were not previously associated with cleft palate (Tables 1 and S2A,B). Four homozygous mutant lines in this set (*Rgl1*, *Slc35d1*, *Slmap*, and *Snx3*) were annotated as having cleft palates by the IMPC team, and we have also detected cleft palates in these mutants (Table 1, Figures 2 and 3). Seven of 484 mutant lines (*Cacna1s*, *Dhcr7*, *Fbxo30*, *Focad*, *Kdelr2*, *Robo1*, and *Ryr1*) were previously associated with cleft palate in mice, while the null mutants of these genes generated by the IMPC did not have cleft palates (Table S2C). Conversely, some of the mutants, even those displaying cleft palates at 100% penetrance (e.g., *Arhgef18*, a novel cleft palate gene), were either missed or not yet annotated by the IMPC as having cleft palates (Table 1). Therefore, although laborious, custom annotations of structural defects in the IMPC mutants are valuable.

**TABLE 1:** Penetrance of cleft palate in the analyzed International Mouse Phenotyping Consortium (IMPC) mutants.

Gene symbol	Embryonic stage	No. of affected embryos	No. of analyzed embryos	Eye defects	IMPC annotations related to craniofacial or eye development
1700003F12Rik	E15.5	1	8	No	No annotation
Acan	E18.5	1	3	No	No annotation
Acvr2a	E15.5	2	6	4 of 9 embryos have eye defects	Anophthalmia, abnormal craniofacial morphology
Acvr2b	E15.5	1	9	No	No annotation
Aebp1	E15.5	1	8	No	No annotation
Anapc15	E15.5	1	8	3 of 8 embryos, but not in the embryo with cleft palate	Anophthalmia, microphthalmia, abnormal craniofacial morphology
Ankrd52	E18.5	1	7	1 of 7; the affected embryos has cleft palate	Microphthalmia, anophthalmia
Ap2b1	E18.5	7	9	No	Cleft palate
Arhgef18	E18.5	4	4	No	No annotation
Bloc1s2	E15.5	2	3	No	Abnormal craniofacial morphology
Bmi1	E15.5	4	8	No	No annotation
Cdk20	E18.5	3	4	4 of 4 have no eyes	Cleft palate, facial cleft, anophthalmia, abnormal facial morphology
Cdk4	E15.5	2	8	1 of 8 embryos has one eye but not CP	Microphthalmia
Cdkn1c	E15.5	7	8	No	No annotation
Celf1	E18.5	1	6	1 of 6 embryos has a small right eye but not CP	No annotation
Cep97	E15.5	8	8	Not examined	Microphthalmia, anophthalmia
Clasp1	E18.5	1	8	No	No annotation
Col11a1	E18.5	6	6	No	No annotation
Col27a1	E15.5	1	4	No	Abnormal craniofacial morphology
Cox7c	E15.5	3	7	No	No annotation
Cramp1	E18.5	1	2	No	Cleft palate
Dnmt3b	E15.5	5	5	No	Abnormal craniofacial morphology, cleft palate
Dpy19l1	E18.5	1	7	No	No annotation
Ednra	E18.5	6	6	No	Abnormal craniofacial morphology
Eif4enif1	E15.5	3	8	No	No annotation
Esrp1	E15.5	3	3	No	Abnormal craniofacial morphology, facial clefting, cleft palate
Eya4	E15.5	2	8	No	No annotation
Fbxo11	E18.5	1	1	No	No annotation
Fgf9	E18.5	2	2	No	Cleft palate
Fgf10s	E18.5	2	3	No	No annotation
Fgf15	E15.5	2	7	No	No annotation
Fndc3b	E15.5	1	8	No	No annotation
Fubp1 ^a^	E15.5	6	6	No	No annotation
Fuz	E15.5	8	8	8 of 8 have no eyes	Abnormal craniofacial morphology
Gdf11	E18.5	11	11	No	Cleft palate, abnormal craniofacial morphology
Inpp5e	E15.5	7	9	Yes, 8 of 9 embryos have no eyes	Abnormal eye morphology, microphtalmia
Jag2	E18.5	2	3	No	Not annotated yet
Kcnj13	E18.5	8	8	No	Cleft palate, abnormal craniofacial morphology
Klf7	E18.5	2	2	No	No annotation
Laptm4b	E15.5	6	8	No	Abnormal eye morphology, abnormal craniofacial morphology
Larp1	E15.5	4	6	No	No annotation, adult heterozygous mice have abnormal kidney morphology
Maf	E15.5	2	5	No	Microphthalmia
Mbd6	E15.5	2	8	No	Microphthalmia, anophthalmia, microcephaly
Memo1	E18.5	2	2	2 of 2 eyes look abnormal	No annotation
Nabp2	E15.5	6	7	No	Abnormal craniofacial morphology
Nmnat2	E15.5	3	4	No	Microphthalmia
Nxn	E18.5	14	16	No	Cleft palate, abnormal craniofacial morphology
Otud6b	E18.5	1	2	No	No annotation
Pccb	E15.5	1	8	Not examined	Microphthalmia, anophthalmia, microcephaly
Pfdn5 ^a^	E15.5	2	2	Yes, 1 of 2 embryos is missing the right eye	Abnormal craniofacial morphology
Pkdcc	E15.5	8	8	No	Abnormal craniofacial morphology
Podxl	E18.5	1	4	1 of 4. The embryo with CP is missing the right eye	No annotation
Pygo2	E15.5	4	6	6 of 6 have abnormal eyes	Anophthalmia, microphthalmia
Rab34	E15.5	3	9	Yes in 3 of 9	Anophthalmia, facial cleft, cleft palate
Rgl1	E15.5	3	6	Yes in 4 of 6	Cleft palate, anophthalmia, microphthalmia
Rsf1	E15.5	5	8	No	No annotation
Runx1t1	E18.5	1	4	No	No annotation
Satb2	E15.5	16	16	No	Abnormal craniofacial morphology
Slc35d1	E18.5	7	7	No	Cleft palate, abnormal facial morphology
Slc7a7	E18.5	2	11	No	No annotation
Slmap	E18.5	8	8	No	Cleft palate, abnormal head size and shape, etc
Smoc1	E18.5	1	2	No	No annotation for embryos
Snx27	E15.5	4	8	No	Abnormal craniofacial morphology
Snx3	E18.5	5	5	Not clear due to severe craniofacial defects	Abnormal craniofacial morphology, anophthalmia, cleft palate
Strn3	E15.5	2	4	No	No annotation
Spop	E15.5	1	11	No	No annotation
Tctn3	E18.5	3	5	Yes in 1 of 5 One embryo with cleft palate is missing right eye	Anophthalmia
Tgfb3	E15.5	8	8	No	No annotation
Tm2d1	E15.5	1	1	No	No annotation
Tm2d2	E15.5	3	3	No	Abnormal craniofacial morphology
Tm9sf3	E15.5	1	8	No	No annotation
Tmem115	E15.5	1	8	Yes in 1 of 8; the embryo with cleft palate is missing the right eye	No annotation
Uggt1 ^a^	E15.5	3	3	Yes in 2 of 3 Could be secondary to abnormal craniofacial dev	Abnormal craniofacial morphology, abnormal facial morphology, anophthalmia
Ugt1a6b	E18.5	1	8	Yes in 2 of 8; two embryos are missing one eye, the embryo with cleft palate has both eyes	Microphthalmia, anophthalmia
Wdfy3	E15.5	1	3	No	Cleft palate, abnormal craniofacial morphology
Wdr45b	E18.5	1	8	No	No annotation

*Note*: Gene names in black ink—known cleft palate mutants; gene names in red ink are novel cleft palate mutants; underlined gene symbols: in these mutants, cleft palate was identified by the IMPC and confirmed by us, but otherwise not studied.

^a^ These mutants appear underdeveloped for the E15.5 stage, and cleft palate in these mutants could have resulted from developmental delay.

Coronal sections displaying defective palatal morphology from 39 novel mutants annotated by us (red in Table 1) and five additional mutants annotated by the IMPC (underlined gene symbols, Table 1) are shown in Figures 2 and 3, with specific embryo information in Tables 2 and 3. Thirty‐eight of the 39 novel genes were not previously associated with cleft palate phenotypes according to both the MGI and the CleftGeneDB databases. The *Col27a1* gene was associated with the cleft palate Mammalian Phenotype (MP) term in the MGI database but not in the CleftGeneDB database. Mutant *Col27a1* alleles were also generated independently of the IMPC; however, palate development was not examined in these mice.^16^ And even though mutations in *Col27a1* were found in some patients with Steel syndrome whose symptoms included cleft palate,^17^ no studies directly linked mutations in *Col27a1* with cleft palate. Therefore, we considered *Col27a1* a novel cleft palate gene. A gene that was initially considered a novel cleft palate gene but ultimately not considered novel in our analysis was *Bmi1*. *Bmi1* was not associated with cleft palate MP terms in the MGI database, but it was present in the CleftGeneDB. Upon further literature review, we found a statement, albeit without supporting image data, that two of six *Bmi1*‐null embryos had cleft palates^18^; therefore, we excluded *Bmi1* from the list of novel cleft palate genes.

**TABLE 2:** Embryos displayed in Figure 2 (E18.5).

Gene name	Embryo number on the IMPC web site
Ankrd52	r36827‐08
Arhgef18	TM1B‐IC/10.4a_6651435
Celf1	552633
Clasp1	CR1488‐170‐4
Dpy19l1	CR1100‐121‐1
Klf7	KLF7‐TM1B‐IC/16.5e_5553718
Otud6b	OTUD6B‐PL‐TM1B‐IC/19.5i_5823697
Podxl	PODxL‐DEL637INS1‐EM1‐B6N‐IC/11.3b_6669674
Runx1t1	TM1B‐FG‐IC/15.1b_6840958
Slc35d1	293068
Slmap	x90132‐2
Snx3	x12093‐01
Tctn3	R27850‐07
Ugt1a6b	561334
Wdr45b	462674

Abbreviation: IMPC, International Mouse Phenotyping Consortium.

**TABLE 3:** Embryos displayed in Figure 3 (E15.5).

Gene name	Embryo number on the IMPC web site
1700003F12Rik	ABBO_K751‐6‐e15.5
Acvr2b	ABTQ_K2032‐5‐e15.5
Aebp1	ACOA_K4705‐6‐e15.5
Anapc15	K47175‐01
Bloc1s2	K44005‐01
Cdk4	ABFE_N2681‐50
Cep97	BL5364‐128‐5
Col27a1	Cr10274‐192‐1
Cox7c	K19278‐02
FGF15	ACPH_K3653‐16
Fndc3b	ACKL_K4596‐4‐e15.5
Fubp1	CR1478‐128‐7
Laptm4b	ABRC_N3482‐7
Larp1	CR1544‐162‐4
Maf	CR10347‐253‐6
Mbd6	Bl2325‐796‐3
Nmnat2	CR10102‐167‐7
Pccb	BL5221‐141‐1
Pfdn5	331,318
Rgl1	CR10147‐195‐2
Rsf1	ABHV_N2260‐14‐e15.5
Snx27	ABQL_K1758‐4‐e15.5
Spop	AAMN_N305‐28
Strn3	K6881‐01
Tm2d1	385,425
Tm2d2	469,012
Tm9sf3	CR1666‐182‐8
Tmem115	ACKR_K3367‐5‐e15.5
Uggt1	CR10532‐171‐1

Abbreviation: IMPC, International Mouse Phenotyping Consortium.

Despite the uniform genetic background (C57BL/6N) used by the IMPC to generate null mutants, the presence of cleft palate was not fully penetrant in the majority of the homozygous null lines in which at least one case of cleft palate was observed (Table 1). Interestingly, even homozygous‐null mutations previously known to be associated with cleft palate phenotype in the literature displayed incompletely penetrant clefting phenotypes in the IMPC mutants (Table 1, e.g., *Slc7a7 and Rab34*). Using a background frequency of cleft palate in mice determined by the IMPC, the occurrence of one case of cleft palate in 11 mutants per line is statistically significant (*p* = .0256). This analysis suggests that even mutants exhibiting cleft palate with low penetrance on a C57BL/6N background play a significant role in palatal development. For example, one of the cleft palate genes identified in our study was *Slc7a7*, and although only 2 of 11 *Slc7a7*‐null IMPC mutants displayed cleft palate (Table 1), published studies have also identified *Slc7a7* as a regulator of palate closure.^19^ Another example is Rab34, which, despite low penetrance, was also confirmed as critical for palatal development in previous studies.^20^ Together, these findings demonstrate our ability to reliably identify genes contributing to cleft palate even at low penetrance, underscoring the value of such mutants in understanding palatogenesis. Although incomplete penetrance of structural defects in the IMPC mutants was noted before, the underlying reasons for phenotypic variability in homozygous‐null mutations on a uniform genetic background are complex and not yet fully understood.^21^

**FIGURE 4:**
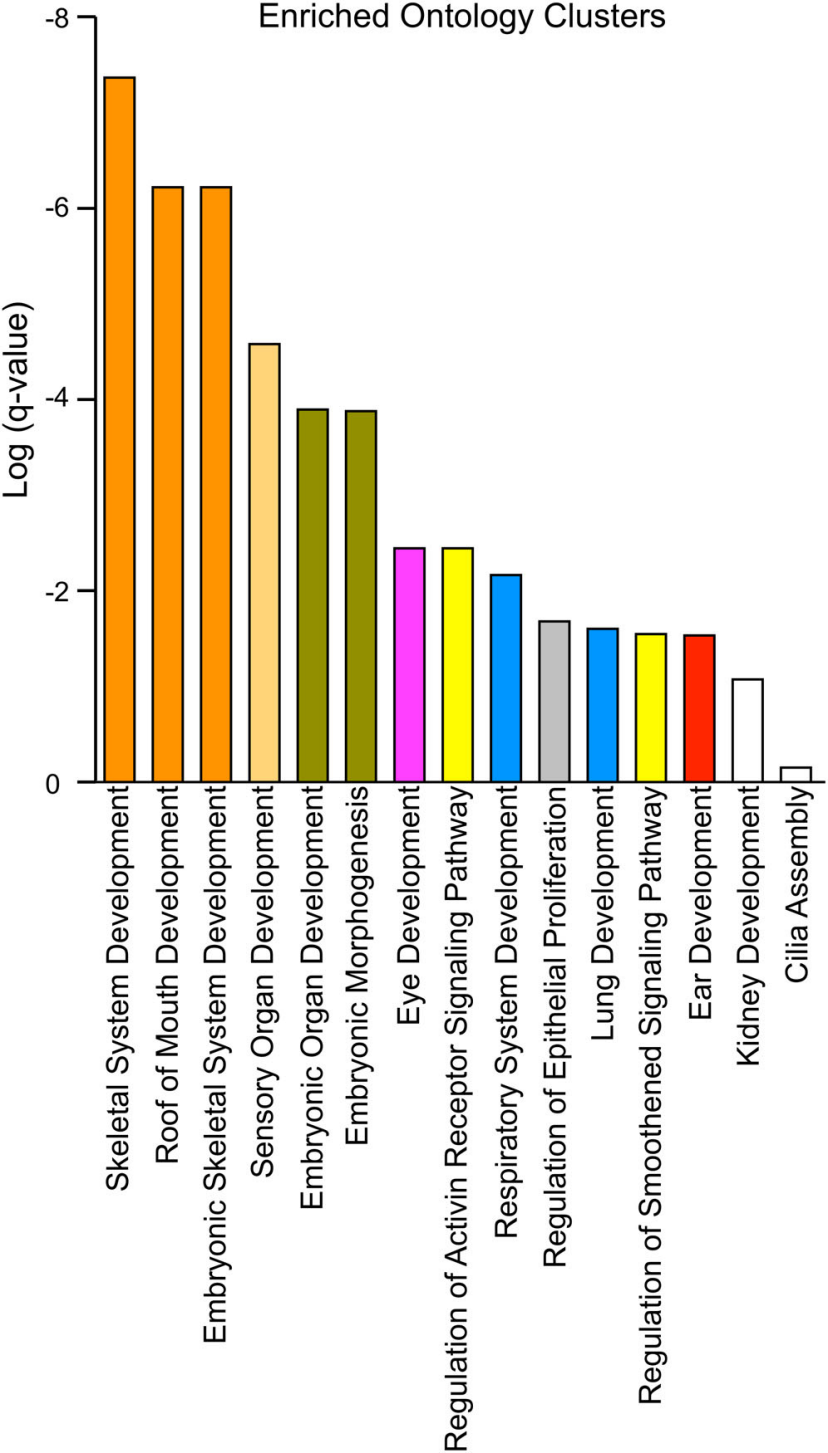
Enriched ontology clusters were determined by using Metascape. See Table S3 for gene lists in each category. *q* is the Benjamini–Hochberg‐adjusted *p*‐value.

Our results suggest that genes whose null mutations result in cleft palate at low penetrance are important for palatogenesis. Therefore, we used the entire list of our annotated cleft palate genes (Table 1) as input for pathway enrichment analysis via Metascape,^22^ a tool that integrates ontology resources such as *Kyoto Encyclopedia of Genes and Genomes (KEGG) Pathway*, *Reactome Gene Sets*, *gene ontology (GO) Biological Processes*, *Canonical Pathways*, *CORUM*, and *WikiPathways*. This analysis identified enriched pathways regulated by cleft palate genes in our screen (Figure 4 and Table S3). Not surprisingly, “Skeletal system development” was the top, most significantly enriched category (Log(*q*) = −7.377, *p* = 4.20E−08, *q* is the *p*‐value adjusted for multiple testing using the Benjamini–Hochberg method). Among the 20 genes in this category, two are novel: *Slc35d1 (identified by the IMPC and confirmed in this study)* and *Col27a1*. NC‐derived cells give rise to the palatal mesenchyme, craniofacial bones, and cartilage; hence, we expected that pathways related to NC development would be enriched in our data set. These pathways included: “Roof of mouth development” (Log(*q*) = −6.230, *p* = 5.89E−07) and “sensory organ development” (Log(*q*) = −4.592, *p* = 2.56E−05). Among the 31 genes in these two categories, *Maf*, *Pfdn5*, *Fndc3b*, and *Col27a1* were novel cleft palate genes (Table S3).

**FIGURE 5:**
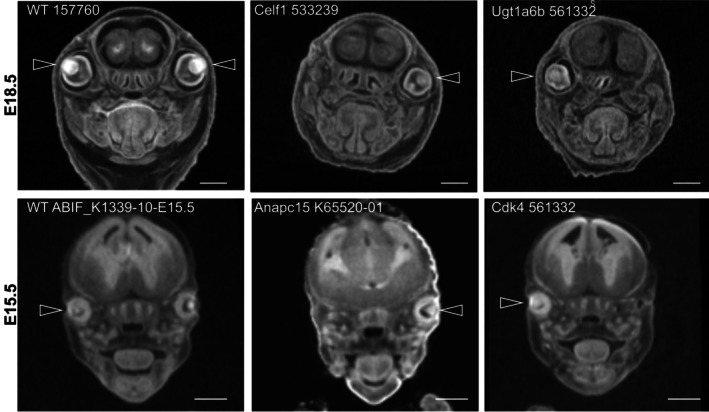
Mutant lines exhibiting eye defects in embryos distinct from those with cleft palate. Snapshots of coronal optical sections from micro‐computed tomography images were taken at eye level. Gene names are followed by International Mouse Phenotyping Consortium (IMPC) embryo numbers. Arrowheads mark eyes. The first image of each row displays coronal slices through the heads of wild type embryos at E18.5 (top row) and E15.5 (bottom row). The mutants shown have only one eye. Additional optical planes and embryos in each line can be viewed on the IMPC website. Scale bars are 1 mm.

Given that NC‐derived cells constitute most of the palatal shelf tissue, the development of organs whose morphogenesis relies on inter‐tissue interactions with NC‐derived cells could also be disrupted in mutants exhibiting cleft palate.^23^ One such organ is the eye, which develops in close proximity to the NC‐derived cells.^24^, ^25^, ^26^ An example of NC‐derived mesenchyme role in influencing eye development is the secretion of the extracellular matrix (ECM) component nidogen by NC‐derived cells, which is important for the proper morphogenesis of the optic cup.^24^ Although ocular defects are among the top 30 birth defects that co‐occur in patients with cleft palate,^27^ the genetic etiology underlying this co‐occurrence is not well understood.^28^, ^29^ The categories “Eye development,” including “Visual system development,” and “camera‐type eye development,” were statistically significantly enriched in our data set (Log(*q*) = −2.455, *p* = 3.5E−02, Figure 4 and Table S3). Therefore, we re‐examined the mutant lines with cleft palate using coronal optical sectioning of the micro‐CT images in the IMPC database and found 18 mutant lines that had both eye and palate defects (Table 1). The eye defects that we phenotyped were limited to morphological anomalies such as anophthalmia (the absence of one or both eyes), microphthalmia (small eyes), and obvious defects in the positioning of the optic cup (Figures 2, 3, and 5, Table S1 for the list of specific embryos exhibiting palatal and eye defects). An independent group reported eye defects in 11 of these mutants,^30^ supporting our phenotype evaluation. IMPC constantly adds lines of imaged mutants to the database, and it is possible that the remaining 7 of 18 lines were not available for viewing when the first group did their analysis.^30^

According to the Centers for Disease Control and Prevention, the prevalence of anophthalmia or microphthalmia in the human population is about 1 in 5100 live births, while the overall frequency of congenital ocular malformations ranges from 0.36% to 4.7%, depending on the study.^31^ In mouse embryos of the C57BL/6N genetic background, the IMPC annotations show higher frequencies of these eye defects. Despite this baseline variation, we observed both cleft palate and eye defects in 18 out of 73 examined mutant lines, a co‐occurrence that is statistically significant (binomial test, *p* < 10^−4^), indicating this association is unlikely to occur by chance. Our phenotyping effort has identified 18 genes involved in regulating the development of both palate and eyes, including eight novel regulators of palate development: *Ankrd52*, *Cox7c*, *Pccb*, *Pfdn5*, *Podxl*, *Rgl1*, *Snx3*, and *Tmem115*.

The NC plays a crucial role in ear development, particularly in the formation and functions of several components of the auditory system.^32^, ^33^ The “Ear development” category was among the statistically significantly enriched ontology clusters in our data set (Log(*q*) = −1.544, *p* = 2.86E−02) (red bar in Figure 4 and Table S3). Among the genes in this category, *Maf* was a novel cleft palate gene. *Maf* encodes a group of transcription factors that can act as transcriptional activators or repressors. Of the four MAFs (*MAFA*, *MAFB*, *c‐MAF*, and *NRL*), mutations in *MAFB* were recently associated with nonsyndromic cleft lip with or without palate.^34^ *MAFB* also regulates the formation of the inner ears, among its other functions, such as the development of pancreatic endocrine cells.^35^ In a recent study of birth defects associated with cleft palate with or without cleft lip, ear impairment was the third most common, after cardiac septation defects.^27^ The enrichment in the “Ear development” category in our data set (Figure 4 and Table S3) demonstrates that our phenotypic annotations and gene set enrichment analyses align with established clinical observations. This consistency underscores the robustness of gene set enrichment analysis in identifying biologically relevant associations.^36^

The “Regulation of epithelial proliferation” category was also statistically significantly enriched biological process (Log(*q*) = −1.691, *p* = 2.04E−02, Figure 4 and Table S3). Proliferation of palatal mesenchyme and epithelium is important for palate development.^4^, ^37^, ^38^ Among the 10 genes in this category, three—*Cdk4*, CUGBP Elav‐like family member 1 (*Celf1*), and *Klf7* were novel cleft palate genes. In the future, it will be interesting to determine whether these genes play a role in regulating cell proliferation during palate formation. Our phenotyping analyses indicated that one of six homozygous *Celf1*‐null mutants imaged by the IMPC exhibited a cleft palate (Figure 2). *Celf1* is an RNA‐binding protein regulating diverse aspects of mRNA biology, including mRNA alternative splicing, stability, and micro RNA (miRNA) processing.^39^ *Celf1* knockout animals were previously generated on a mixed C57BL6‐129Sv background, and although ~50% of the knockout animals did not survive past Day 10 after birth, palatal development was not examined.^40^ Celf1 protein binds guanine and uracil (GU)‐rich sequences in introns as well as in 5′‐and 3′‐UTRs of mRNAs. The role of *Celf1* in palate development could be related to its ability to regulate the expression of *Snail1*,^41^ a known contributor to palatogenesis.^42^ However, further mechanistic studies are needed to elucidate how *Celf1*, along with *Cdk4* and *Klf7*, influences palate development.

The enrichment of genes in the “Respiratory system development” pathway was particularly intriguing, as it contained the highest number of novel cleft palate‐associated genes identified in our screen (blue bars mark both “Respiratory system development” and “Lung development” clusters in Figure 4). Among the 29 genes in the “Respiratory system development” ontology cluster, nine were novel cleft palate genes: *Cdk4*, *Col27a1*, *Fgf15*, *Fndc3b*, *Maf*, *Pfdn5*, *Rgl1*, *Runx1t1*, and *Snx3* (Table S3). Col27a1 (type XXVII collagen), an evolutionarily conserved ECM protein and member of the fibrillar collagen family,^43^ exhibits an unusual domain structure and developmentally restricted expression, suggesting critical roles in morphogenesis.^44^ Mutations in *Col27a1* cause Steel syndrome, a rare autosomal recessive skeletal disorder.^17^, ^45^, ^46^ While one pair of affected twins with Steel syndrome presented with cleft palate^17^, this phenotype was absent in other Steel syndrome patients and in *Col27a1*‐null mice on a mixed genetic background.^16^ In our study, one of four *Col27a1*‐null IMPC mutants exhibited cleft palate—a statistically significant finding (*p* = .0094), albeit with low penetrance. Given that other genes in this pathway (*Fgf9*, *Fgf10*, *Tgfβ3*, and *Pkdcc*) are established regulators of both lung and palate development,^47^, ^48^, ^49^, ^50^, ^51^ and that *Col27a1* is expressed in the lung,^52^ it is possible that *Col27a1* may also function in these processes.

The enrichment in the “Kidney development” category (Log(*q*) = −1.085, *p* = 8.22E−02) comprising the genes *Acvr2b*, *Ap2b1*, *Cdkn1c*, *Ednra*, *Fgf10*, *Gdf11*, *Pygo2*, was also consistent with prior studies that identified genes involved in both kidney and palate development.^53^, ^54^ Published studies indicate that *Pygo2*, a component of the canonical Wnt signaling pathway, and *Gdf11* and *Acvr2b*, components of the activin signaling pathway, are required for the development of both the palate and the kidney,^53^, ^54^, ^55^ although the mechanism by which *Pygo2* mutants regulate palate development is unknown. The enrichment in this category and prior literature suggests the existence of common regulatory mechanisms mediating the development of the kidney and the palate. Further investigation of these shared pathways may identify molecular nodes susceptible to disruption in congenital anomalies.

In addition to biological processes, our data set was enriched for two pathways known to be involved in palatal development, “Regulation of activin receptor signaling pathway” (Log(*q*) = −2.455, *p* = 3.51E−03) and “Regulation of smoothened signaling pathway” (Log(*q*) = −1.559, *p* = 2.76E−02), which were statistically significantly enriched (yellow bars in Figure 4). This finding aligns with established literature underscoring the critical role of these pathways in palatogenesis.^4^, ^11^, ^53^, ^56^, ^57^ Interestingly, among the 13 genes in the “Regulation of activin receptor signaling pathway,” three were novel: *Fndc3b*, *Arhgef18*, and *Fgf15*. Notably, homozygous‐null *Arhgef18* mutants exhibited complete penetrance of cleft palate (Table 1). *Arhgef18* encodes a RhoA guanine nucleotide exchange factor (GEF), also known as p114RhoGEF, that regulates RhoA activation and actomyosin contractility in multiple contexts.^58^, ^59^, ^60^, ^61^ Activation of Rho and actomyosin contractility is essential to form a continuous palatal tissue.^62^ Future conditional mutagenesis and mechanistic studies are necessary to determine how *Arhgef18* regulates palatal development. This is particularly relevant given recent findings linking *Arhgef18* mutations to sporadic cleft palate in humans.^63^

In addition to *Arhgef8*, we identified another mutant line—a null mutation in the *Tctn3* (tectonic family member 3) gene—that exhibited a penetrant cleft palate phenotype (Figure 2 and Table 1). Although 100% penetrant, the cleft palate phenotype was missed by the IMPC annotation pipeline, indicating that manual, investigator‐initiated annotations are important. In humans, mutations in *Tctn*3 are associated with the Mohr‐Majewski Syndrome, and a cleft palate was found in one case.^64^ However, palatal development was not examined in *Tctn3*‐null mutants generated before the IMPC screen^65^; therefore, before our phenotyping screen, it was not clear whether *Tctn3* was required for the development of the palate. The *Tctn3* gene encodes a membrane protein that localizes to the primary cilium and is essential for the activation of Shh signaling.^65^, ^66^ Ablation of *Tctn3* resulted in defective ciliogenesis.^65^ Interestingly, ciliopathies (defects resulting in dysfunctional primary cilia) are common causes of craniofacial defects and underly several craniofacial syndromes, such as Joubert syndrome [Online Mendelian Inheritance in Man] (OMIM 213300), Meckel‐Gruber syndrome (OMIM 249000), Ellis‐van Creveld syndrome (OMIM 225500), oral–facial–digital syndromes types IX (OMIM 258865) and XIV,^67^, ^68^ and Weyers acrofacial dysostosis (OMIM 193530).^69^, ^70^ Consistent with this, our gene set enrichment analysis showed that the “Cilium assembly” category was detected in our data set (Figure 4 and Table S3). This category included the following genes: *Tctn3, Cep97, Inpp5e, Fuz*, and *Clasp1* (Table S3), among which *Tctn3*, *Cep97*, and *Clasp1* are novel cleft palate genes identified in our screen (Figures 2 and 3). Cep97 localizes at the centrioles and regulates cilia assembly.^71^ *Clasp1* encodes a plus‐end microtubule‐binding protein regulating the microtubule dynamics,^72^, ^73^ an essential process for the biogenesis of cilia.^74^ Cilia are essential for the regulation of Shh and smoothened signaling,^75^ and our screen identified three additional genes (*Tctn3*, *Cep97*, and *Clasp1*) with roles in cilia, microtubule dynamics, and palatal development.

In summary, our studies not only identify novel genes involved in palatal development but also provide new insights into the etiology of rare congenital defects affecting both the eyes and palate. The screen of IMPC mutants revealed 44 previously unrecognized genes regulating palatal development. Further analysis of additional IMPC mutants will yield deeper insights into the biological processes and pathways critical for palatal development and malformations. Another value of such analyses lies in identifying unanticipated organs and pathways that could be affected alongside the palate in patients, allowing for a more thorough clinical follow‐up and treatment of associated co‐morbidities.

## 3 EXPERIMENTAL PROCEDURES

### 3.1 Image analysis

To identify novel genes regulating the development of the secondary palate, we screened E15.5 and E18.5 homozygous‐null mutant embryos generated and imaged by the IMPC: https://www.mousephenotype.org.^76^ Coronal optical sections of micro‐CT images were analyzed using the tools embedded within the IMPC website to identify embryos with defective palates. Embryos at E18.5 were preferred for analysis due to the palate being fully fused at this stage in wild‐type mouse embryos. E15.5 embryos were also analyzed. In E15.5 controls, the anterior‐most and the posterior palate were often not yet fused. Therefore, cleft palate was only marked in those E15.5 mutants in which the palatal shelves were not fused throughout the entire length of the palate. Mutants containing cleft palates were also evaluated for eye defects, a category suggested by gene set enrichment analyses using Metascape (see Results and Discussion section).

### 3.2 Statistical analysis

The IMPC has determined the occurrence of cleft palate in wild‐type embryos isolated at two developmental stages, E15.5 and E18.5. At E15.5, cleft palate was observed in 3 out of 832 female embryos and 0 out of 862 male embryos. At E18.5, it was observed in 5 out of 980 female embryos and 2 out of 1044 male embryos (https://www.mousephenotype.org/data/supporting-data?mgiGeneAccessionId=MGI:2140361&mpTermId=MP:0000111&alleleAccessionId=MGI:5692775&lifeStageName=E18.5 and https://www.mousephenotype.org/data/supporting-data?mgiGeneAccessionId=MGI:1917326&mpTermId=MP:0000111&alleleAccessionId=MGI:6276892&lifeStageName=E15.5). Putting a cursor over the bars in the graph displays defect frequencies.

Among the identified novel mutants with cleft palate, *Spop*‐null homozygotes showed the lowest penetrance, with only 1 of 11 embryos affected. Using combined male and female data from the E15.5 time point and Fisher's exact test, the two‐tailed *p* value for the null hypothesis equals .0256, indicating that even at the frequency of 1 cleft palate in 11 embryos, it is statistically significant.

The IMPC also evaluated the frequency of anophthalmia and microphthalmia at E15.5 and E18.5 in wild‐type embryos on the C57BL/6N genetic background used to generate mutants. At E15.5, anophthalmia was observed in 15 out of 832 female embryos and 2 out of 862 male embryos; and microphthalmia was observed in 6 out of 832 female and 2 out of 862 male embryos. At E18.5, anophthalmia was observed in 15 out of 980 female embryos and 2 out of 1044 male embryos, and microphthalmia was observed in 27 out of 980 female embryos and 2 out of 1044 male embryos. The overall frequency of co‐occurrence of cleft palate and anophthalmia or microphthalmia by chance alone is 11.43 × 10^−5^ at E15.5 and 78.6 × 10^−6^ at E18.5. The probability that cleft palate and eye defects co‐occur by chance is 2.61 × 10^−5^ at E15.5 and 78.6 × 10^−6^ at E18.5.

### 3.3 Identification of novel cleft palate genes

To find which of the cleft palate mutations were novel, we queried Mammalian Phenotype ontology terms associated with the genes on our list (Table 1). To do so, we queried the list of genes in Table S1 against mammalian phenotype terms (https://www.informatics.jax.org/batch, Tables S2A–C). In addition, we compared the genes on our list with those in a cleft gene database (CleftGeneDB, https://bioinfo.uth.edu/CleftGeneDB/). We also manually queried each gene in PubMed and Google. Genes not previously identified as playing a role in palatal development in the mouse knockout studies were considered novel (Table 1, red). Underlined gene symbolsmark cleft palate mutants found only in IMPC data, with no other published records (Table 1).

### 3.4 Pathway enrichment analyses

We used Metascape^22^ to identify enriched biological processes and pathways (Figure 5 and Table S3).

## Supporting information


**Table S1.** Cleft palate and eye defects in IMPC mutants.
**Table S2**. Mouse genome informatics batch report.
**Table S3**. Metascape gene set enrichment results.

## Data Availability

All the data are publicly available.
